# Supplementary material for: Robust singlet dimers with fragile ordering in two-dimensional honeycomb lattice of Li2RuO3
Source: Sci Rep. 2016 May 4;6:25238. doi: 10.1038/srep25238 (PMC4855220; doi:10.1038/srep25238)
Supplement: Supplementary Information [file srep25238-s1.pdf]

Supplementary Information

Robust singlet dimers with fragile ordering in two-dimensional honeycomb lattice of Li_2RuO_3

Junghwan Park*[1], Teck-Yee Tan*[2], D. T. Adroja[3,4,$], A. Daoud-Aladine[3], Seongil Choi[1,2], Deok-Yong Cho[1,2], Sang-Hyun Lee[1,2], Jiyeon Kim[1], Hasung Sim[2,5], T. Morioka[6], H. Nojiri[6], V. V. Krishnamurthy[7], P. Manuel[3], M. R. Lees[8], S.V. Streltsov[9,10], D.I. Khomskii[11,$], and Je-Geun Park[1,2,5,$]

[1] Center for Strongly Correlated Materials Research, Seoul National University, Seoul 08826, Korea
[2] Center for Correlated Electron Systems, Institute for Basic Science, Seoul 08826, Korea
[3] ISIS Facility, Rutherford Appleton Laboratory, Didcot OX11 0QX, United Kingdom
[4] Highly Correlated Matter Research Group, Physics Department, University of Johannesburg, Auckland Park 2006, South Africa
[5] Department of Physics and Astronomy, Seoul National University, Seoul 08826, Korea
[6] Institute for Materials Research, Tohoku University, Sendai 980-8577, Japan
[7] Department of Physics and Astronomy, George Mason University, Fairfax, VA 22030-4444, USA
[8] Department of Physics, University of Warwick, Coventry CV4 7AL, United Kingdom
[9] Institute of Metal Physics, Ekaterinburg 620041, Russia
[10] Department of Theoretical Physics and Applied Mathematics, Ural Federal University, Ekaterinburgh 620002, Russia
[11] II Physikalisches Institut, University of Koeln, 50937 Koeln, Germany

* Equally contributed
$ Correspondence to J.G.P. [email: jgpark10@snu.ac.kr], D.I.K. [email: khomskii@ph2.uni-koeln.de] & D.T.A. [email: devashibhai.adroja@stfc.ac.uk]

SI Table 1: Summary of the structure analysis of the Li_2RuO_3 sample with the data presented in Fig. SI1.

Sample	LRO3	
Temperature (K)	300	550
Space group	$P2_1/m$	$C2/m$
a (Å)	4.9392(3)	5.0461(2)
b (Å)	8.7692(5)	8.7537(3)
c (Å)	5.8881(3)	5.9287(2)
β (°)	124.4446(25)	124.5277(31)
V (Å^3)	210.333(21)	215.752(15)
Ru–Ru (Å)	2.605(4)<br>3.012(4)<br>3.052(2)	2.934(2)<br>2.907(1)<br>2.907(1)

SI Table 2: Summary of the detailed final sintering conditions for the six Li_2RuO_3 samples with different doping ratio (x) between Li and Ru atoms on the Ru honeycomb lattice.

Sample Name	Mixing ratio (x)	Starting materials	Sintering condition
**LRO1**	$x \approx 0$	Li_2CO_3 (10 mol % excess) + Ru	1000 °C for 98 h (pellet placed in alumina crucible)
**LRO2**	$x \approx 0.07$	Li_2CO_3 (10 mol % excess) + RuO_2	1000 °C for 48 h (pellet placed in alumina crucible)
**LRO3**	$x \approx 0.11$	Li_2CO_3 (10 mol % excess) + RuO_2	1000 °C for 92 h (pellet placed in alumina crucible)
**LRO4**	$x \approx 0.14$	Li_2CO_3 (10 mol % excess) + Ru	1100 °C for 198 h (powder inserted in Pt tube, sealed tightly)
**LRO5**	$x \approx 0.16$	Li_2CO_3 (10 mol % excess) + RuO_2	1000 °C for 48 h (pellet placed in alumina crucible)
**LRO6**	$x \approx 0.22$	Li_2CO_3 (20 mol % excess) + RuO_2	1000 °C for 48 h (pellet placed in alumina crucible)

SI Table 3: Summary of the structural data for the six Li_2RuO_3 samples with different doping ratio (*x*). The powder x-ray diffraction patterns are shown in Fig. SI3. A comparison of the FWHM(full width at half maximum) of the (001) Bragg peak for all six samples with that for Li_2TiO_3 indicates that all our samples form with a very high level of crystallinity.

Sample	FWHM (degree)	*a* (Å)	*b* (Å)	*c* (Å)	β (°)	V (Å^3)
LRO1	0.148(6)	4.9245(3)	8.7665(6)	5.8880(4)	124.382(1)	209.272(23)
LRO2	0.164(5)	4.9217(2)	8.7788(4)	5.8938(2)	124.352(2)	210.238(15)
LRO3	0.168(8)	4.9334(3)	8.7790(5)	5.8946(4)	124.406(3)	210.636(22)
LRO4	0.174(6)	4.9426(2)	8.7818(3)	5.8934(3)	124.435(2)	210.988(16)
LRO5	0.173(7)	4.9440(3)	8.7826(4)	5.8957(4)	124.450(3)	211.104(21)
LRO6	0.172(6)	4.9658(4)	8.7764(7)	5.8946(5)	124.540(5)	211.612(29)
Li_2TiO_3	0.166(6)	5.0713(4)	8.7882(6)	5.0909(3)	109.294(8)	214.143(26)

SI Table 4: Summary of the structural analysis of two Li_2RuO_3 samples with different doping ratio (x) from high-resolution neutron diffraction data taken in the high-temperature phase (see Fig. SI 4).

Sample	LRO2	DTA
Mixing ratio (x)	0.066(2)	0.129(4)
Temperature (K)	550	580
Space group	*C*2/*m*	*C*2/*m*
a (Å)	5.05099(4)	5.06358(7)
b (Å)	8.76479(8)	8.75096(11)
c (Å)	5.94204(7)	5.93822(9)
β (°)	124.530(1)	124.614(1)
V (Å^3)	216.715(4)	216.556(5)
Ru–Ru (Å)	2.934(4)<br>2.911(2)	2.933(6)<br>2.914(3)

SI Fig. 1: Structure refinement of the Li_2RuO_3 sample (LRO3) below and above the transition temperature using high-resolution XRD (X-ray diffraction) data with the summary given in Table SI 1. The green ticks indicate the position of the Bragg peaks and the blue lines at the bottom show the difference curves. The insets show the enlarged pictures of the data at low angles.

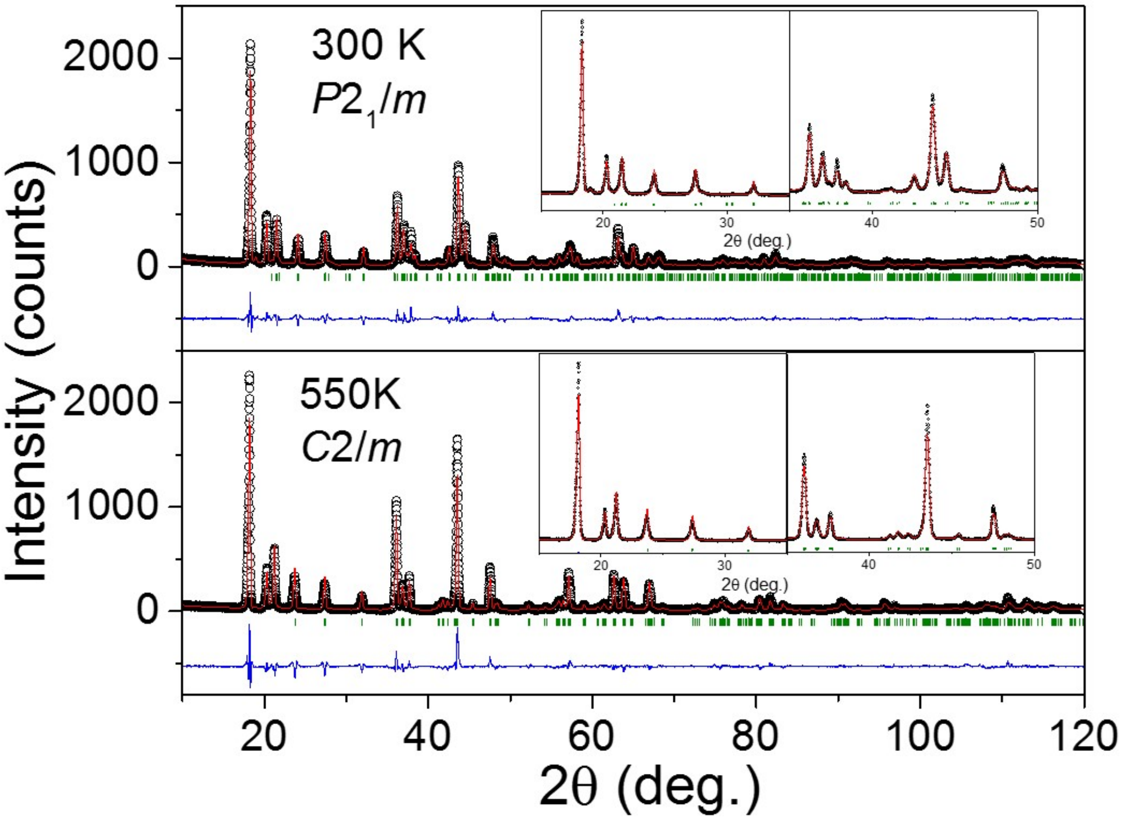

SI Fig 2: High resolution x-ray diffraction data of Li_2RuO_3 and Li_3RuO_4. The symbols represent the data while the red line is the refinement results. The green ticks indicate the position of the Bragg peaks and the blue lines at the bottom show the difference curves.

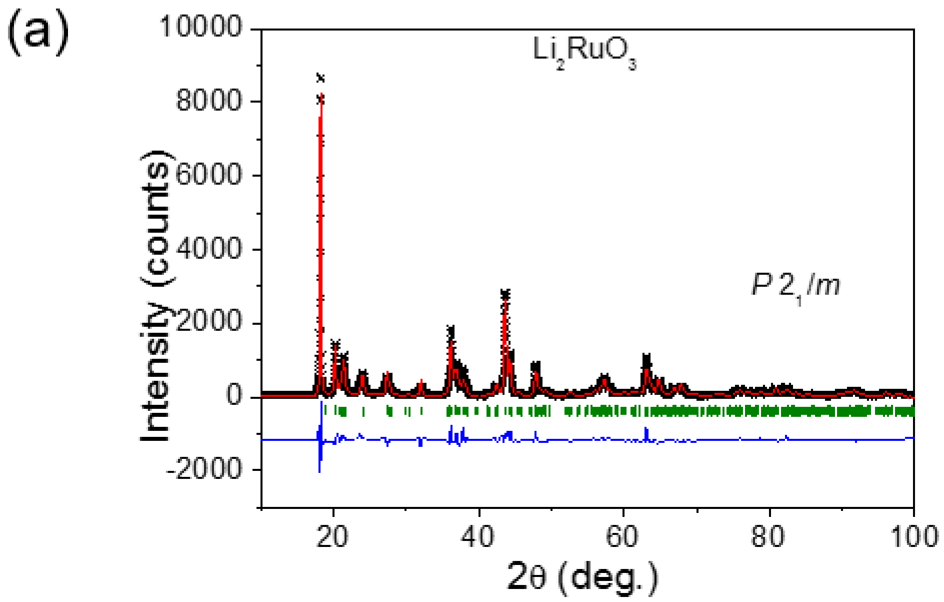

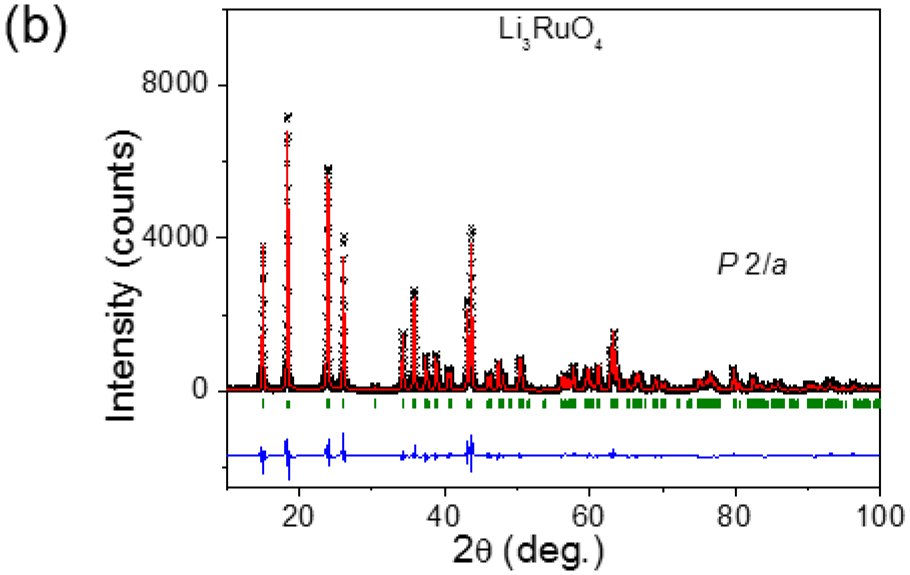

SI Fig 3: X-ray diffraction data of six Li_2RuO_3 samples with different doping ratio (x). The synthesis methods are summarized in Table SI 2. The shaded area indicates where we expect to see the superlattice peak of the $P2_1/m$ phase.

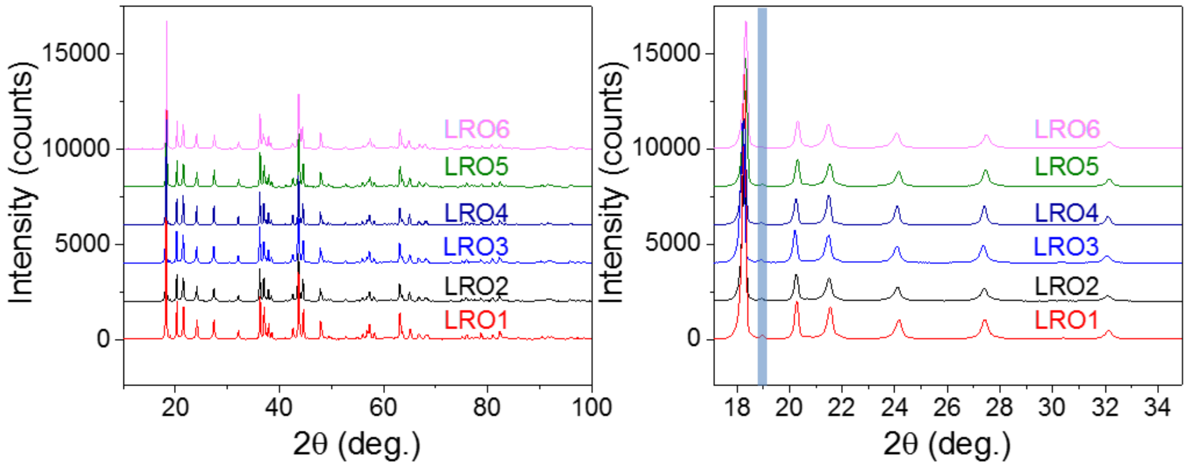

SI Fig 4: Neutron diffraction data taken at high temperature for two Li_2RuO_3 samples: LRO2 (with x = 0.07) and the DTA (with x = 0.13) samples. The green ticks indicate the position of the Bragg peaks and the blue lines at the bottom show the difference curves. A summary of the refinement results is given in Table SI4.

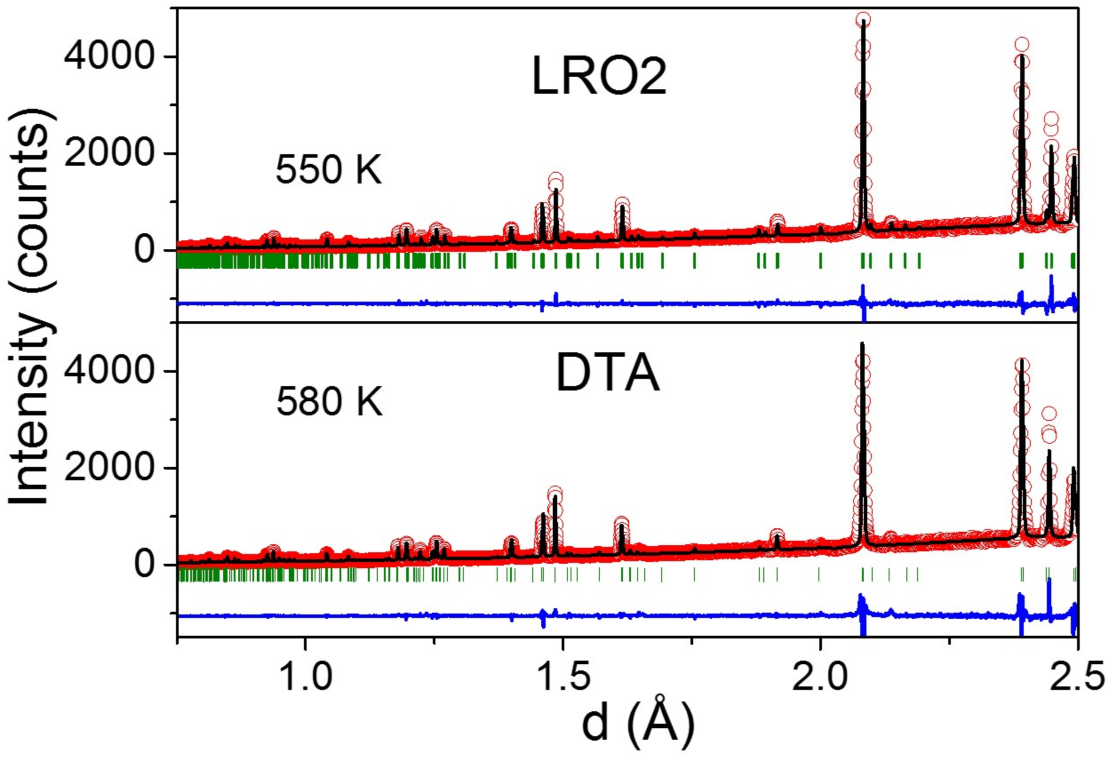

SI Fig 5: (a) Colour plot of scattering intensity as a function of energy transfer & Q and (b) Q-integrated energy cut for the Q range from 0 to 1.5 $Å^{-1}$, measured by an inelastic neutron scattering technique on the Li_2RuO_3 sample: LRO5 (with x = 0.16). (c) The heat capacity and (d) susceptibility data shown in the bottom two panels demonstrate that this new LRO5 sample (2nd batch) synthesized for the inelastic neutron experiments has almost the same bulk properties as the LRO5 sample (1st batch) that was used for the bulk measurements shown in Figs. 2 & 3.

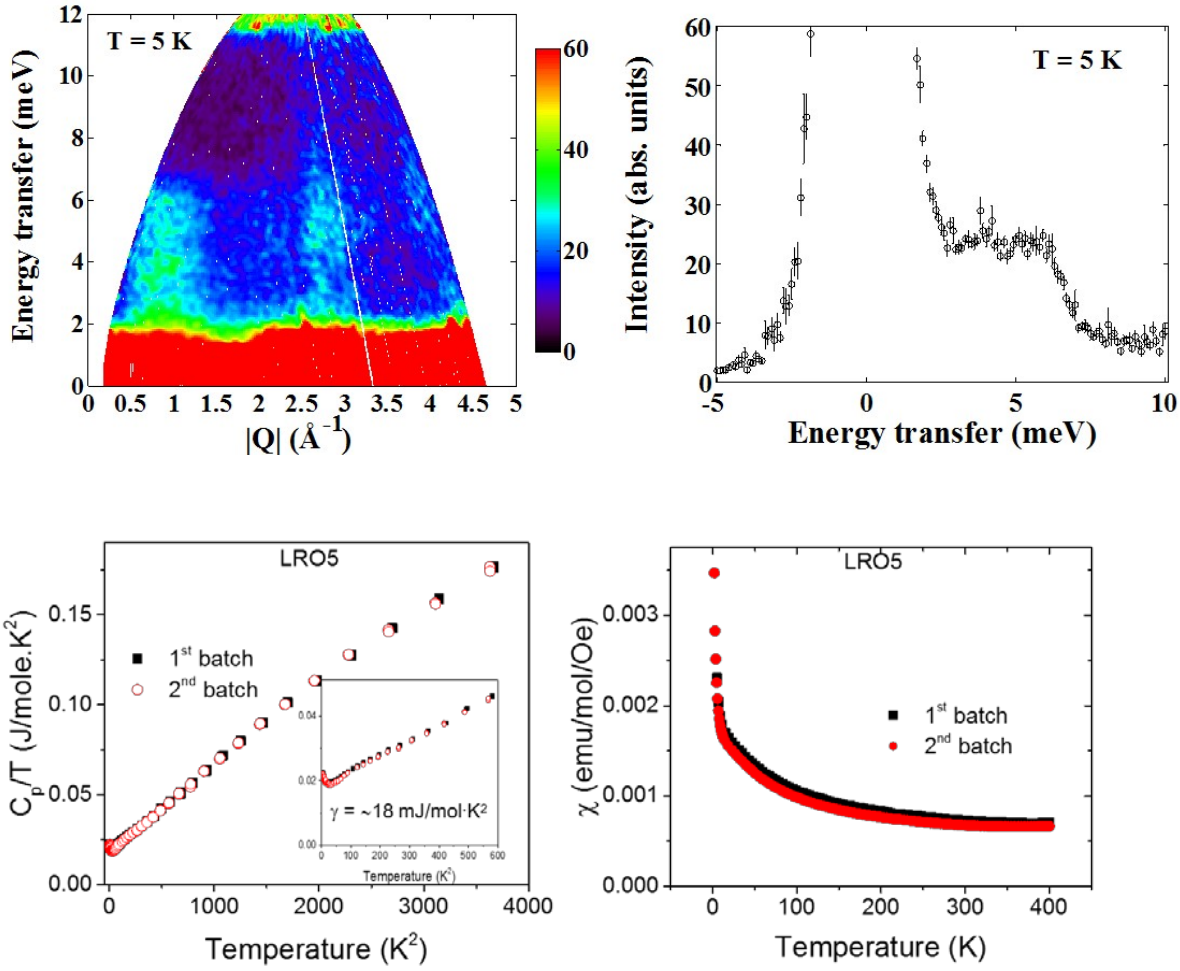

SI Fig 6: Temperature dependence of the low-energy magnetic excitations measured on two Li_2RuO_3 samples: LRO2 (x = 0.07) and DTA (x = 0.13) with an incident energy Ei=13 meV at MERLIN.

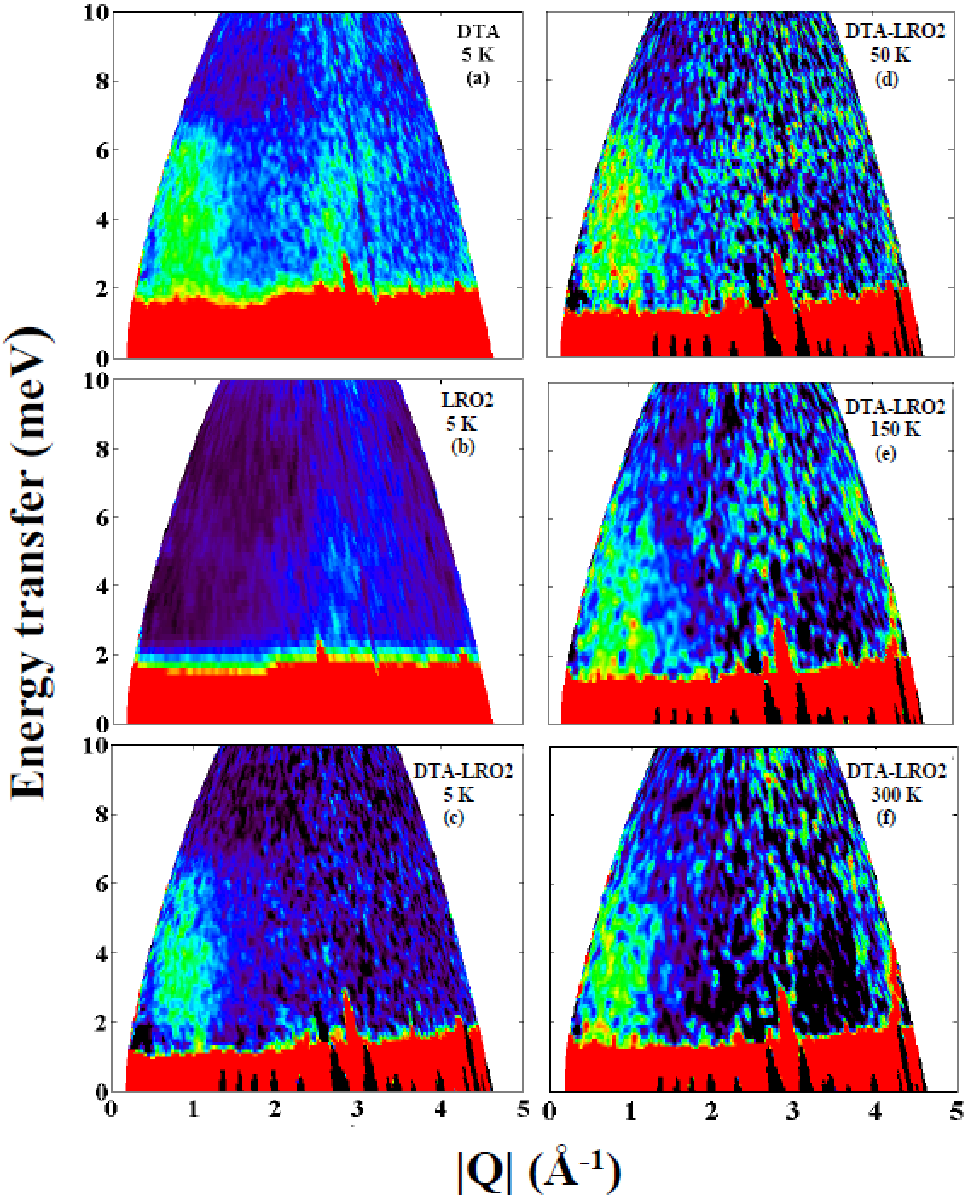

SI Fig 7: Energy versus momentum plot of (a) the low-energy magnetic excitations and (b) their Q-dependence of the Li_2RuO_3 DTA (x = 0.13) sample. The solid lines in (b) show the fit to the isolated dimer model using different values of correlation lengths while the dotted line represents the background.

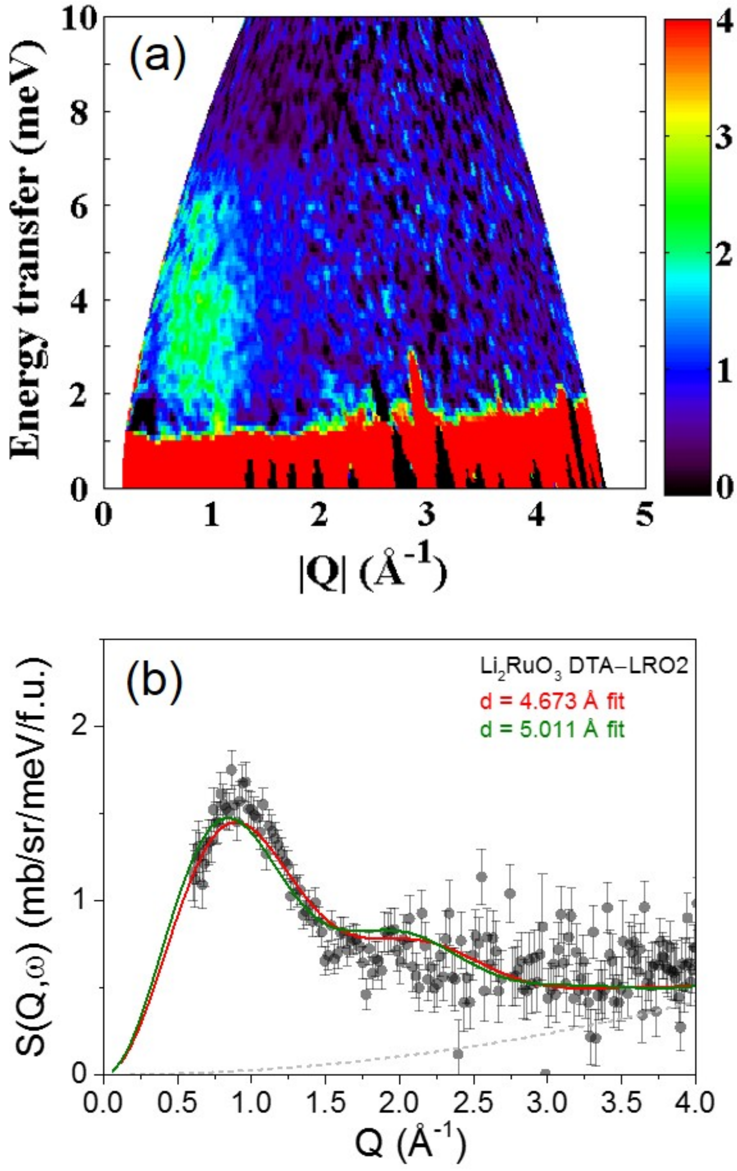

SI Fig 8: Comparison of the bulk susceptibility with the uniform susceptibility calculated using the inelastic neutron scattering data for the two Li_2RuO_3 samples (LRO2 with x = 0.07 & DTA with x = 0.13).

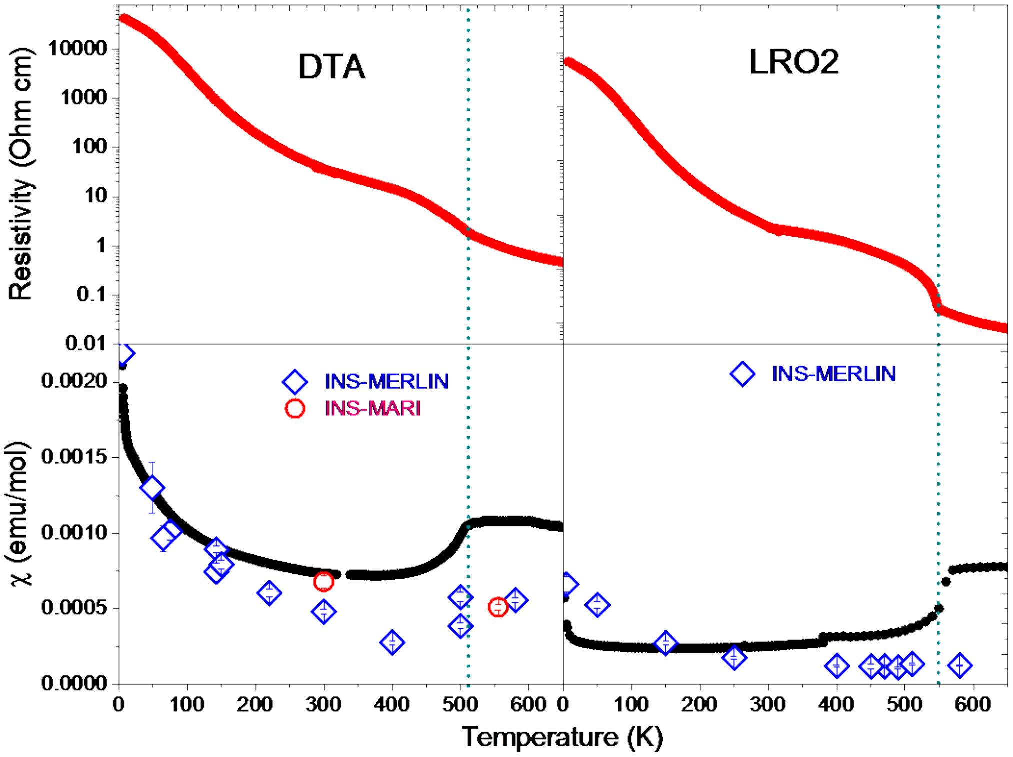

SI Fig 9: A plot of the electronic contribution to the heat capacity (γ) versus the low-temperature value of the susceptibility (χ_0) for the Li_2RuO_3 systems with different levels of disorder (*x*) compared with those for other heavy fermion systems. Data for the other heavy fermion systems were taken from Ref. SI 1. This plot shows that the Sommerfeld-Wilson ratio: $R = \frac{4\pi^2 k_B^2 \chi_0}{3(g\mu_B)^2\gamma}$, is found to be less than one for our Li_2RuO_3 materials with different levels of disorder (*x*).

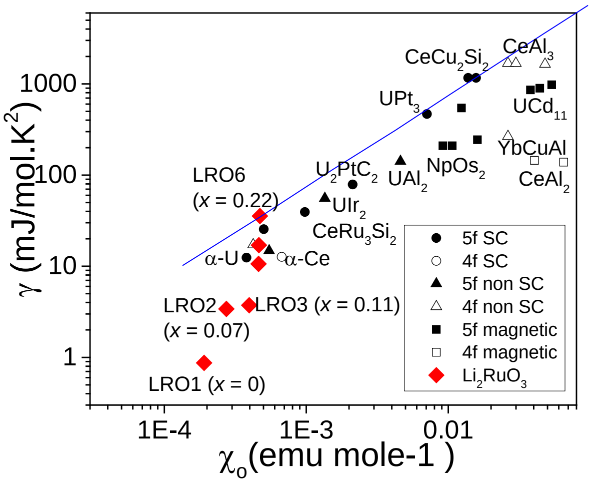

SI Fig 10: A plot of the difference between spin-densities in normal and Li/Ru interchanged Li_2RuO_3. By colours we show different signs of this difference. Ru ions are grey balls, connected by thick line if they form a dimer. O and Li are not shown for simplicity. One can see one broken dimer (bottom left side) and the hexagon with Ru in its centre (top right side).

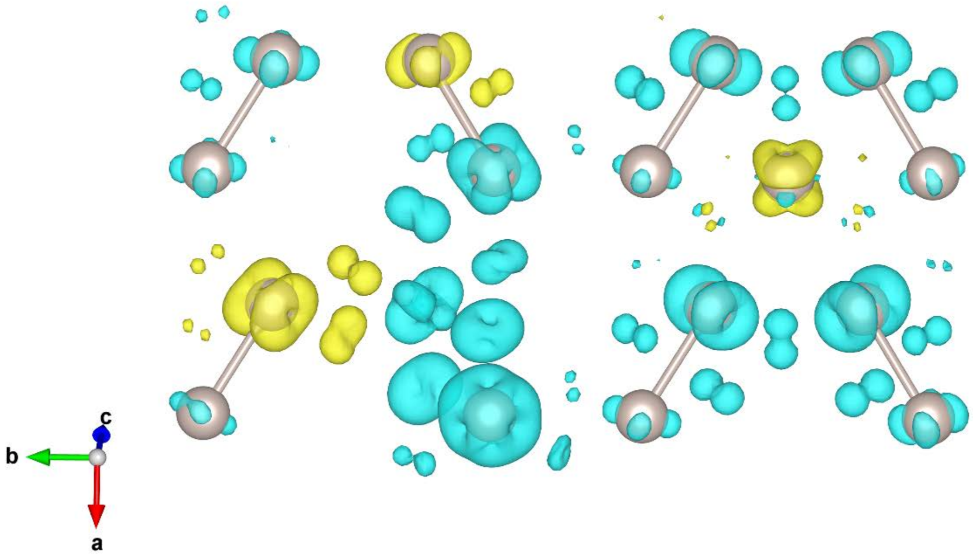

[SI 1] Jones, B.A. Handbook of Magnetism and Advanced Magnetic Materials Vol. 1 (eds Kronmuller, H. and Parkin, S.) Chapter 2. Kondo effect (John Wiley & Sons, 2007).
